# Enhanced removal of perfluorooctanoic acid with sequential photocatalysis and fungal treatment

**DOI:** 10.1007/s11356-023-28588-5

**Published:** 2023-07-20

**Authors:** Mohd Faheem Khan, Jhimli Paul Guin, Ravindranathan K. Thampi, James A. Sullivan, Cormac D. Murphy

**Affiliations:** 1 School of Biomolecular and Biomedical Science, University College Dublin, Belfield , Dublin 4 Ireland; 2 School of Chemical and Bioprocess Engineering, University College Dublin, Belfield, Dublin 4 Ireland; 3 School of Chemistry, University College Dublin, Belfield, Dublin 4 Ireland

**Keywords:** Biodegradation, *Cunninghamella*, Fluoride, Fluorometabolite, PFAS, Bismuth oxyiodide, Photocatalysis

## Abstract

In this paper, we report the degradation of perfluorooctanoic acid (PFOA), which is a persistent contaminant in the environment that can severely impact human health, by exposing it to a photocatalyst, bismuth oxyiodide (BiOI), containing both Bi_4_O_5_I_2_ and Bi_5_O_7_I phases and a fungal biocatalyst (*Cunninghamella elegans*). Individually, the photocatalyst (after 3 h) and biocatalyst (after 48 h) degraded 35–40% of 100 ppm PFOA with 20–30% defluorination. There was a marked improvement in the degree of degradation (90%) and defluorination (60%) when PFOA was first photocatalytically treated, then exposed to the fungus. GC- and LC–MS analysis identified the products formed by the different treatments. Photocatalytic degradation of PFOA yielded short-chain perfluorocarboxylic acids, whereas fungal degradation yielded mainly 5:3 fluorotelomer carboxylic acid, which is a known inhibitor of cytochrome P450-catalysed degradation of PFAS in *C. elegans*. The combined treatment likely resulted in greater degradation because photocatalysis reduced the PFOA concentration without generating the inhibitory 5:3 fluorotelomer carboxylic acid, enabling the fungus to remove most of the remaining substrate. In addition, new fluorometabolites were identified that shed light on the initial catabolic steps involved in PFOA biodegradation.

**Supplementary Information:**

The online version contains supplementary material available at 10.1007/s11356-023-28588-5.

## Introduction

Per- and poly-fluorinated alkyl substances (PFAS) are organic compounds in which most or all of the hydrogens usually bonded to carbon atoms are substituted by fluorine. Their properties of heat, stain, and water resistance mean that they are used in a wide range of products including surfactants, fire-fighting foams, non-stick cookware (Teflon), and stain-resistant fabrics (Glüge et al. 2020). They are highly persistent in the environment and are widespread pollutants (Kurwadkar et al. 2022). Perfluorooctanoic acid (PFOA) and perfluorooctane sulfonate (PFOS) are the focus of much current attention as humans have been exposed to them from a variety of sources (food, water, clothing, etc.), and these compounds have been detected in human milk, blood, and cerebrospinal fluid (Han et al. 2023; Hu et al. 2023; Liu et al. 2023). The health implications are severe, with diseases including certain cancers (kidney and testicular), pregnancy-induced high blood-pressure, ulcerative colitis, and high cholesterol, being linked with PFAS exposure (Sunderland et al. 2019). Consequently, there has been a considerable research effort in recent years to develop a range of chemical, physical, and biological methods to remediate PFAS contamination (Dickman and Aga 2022; Leung et al. 2022). Meegoda et al. (2022) compared different physicochemical methods of PFAS destruction: electrochemical oxidation, plasma, photocatalysis, sonolysis, supercritical water, and incineration, and observed that all could degrade PFAS to varying degrees, and all methods had distinctive disadvantages. In many cases, the drawbacks had environmental consequences, such as the requirement for heavy metals (electrochemical oxidation), high energy consumption (sonolysis), and the formation of toxic intermediate products and by-products (supercritical water oxidation, incineration).

Biological removal of pollutants is desirable on the basis of cost and environmental sustainability; however, fluorinated compounds can be difficult to biodegrade owing to the stability of the carbon–fluorine bond and the dearth of enzymes that have evolved to directly attack it (Seong et al. 2019). Fluorine’s small van der Waals radius means that fluorinated compounds do not encounter steric barriers in enzyme active sites and are often accepted as substrates. Thus, catabolism of organofluorine compounds can occur along established catabolic pathways either leading to unmetabolizable fluorinated dead-end products or resulting in spontaneous fluoride elimination from an unstable intermediate (Murphy 2010; Bygd et al. 2021). Biodegradation of PFAS has been studied in mixed and pure cultures, with most studies concentrating on fluorotelomer alcohols, such as 6:2 FTOH (Zhang et al. 2022). Bacteria and fungi can degrade these compounds yielding a range of fluorometabolites such as 5:3 fluorotelomer carboxylic acid (5:3 FTCA), 6:2 fluorotelomer unsaturated carboxylic acid (6:2 FTUCA), 5:2 ketone, 5:2 secondary alcohol, and shorter perfluorocarboxylic acids (Kim et al. 2014; Tseng et al. 2014; Liu et al. 2010; Khan and Murphy 2022).

In contrast to the biodegradation of fluorotelomer alcohols, there are very few studies reporting the microbial degradation of PFOA. Earlier studies suggested that the compound was microbiologically inert (Liou et al. 2010); however, Yi et al. (2016) isolated a *Pseudomonas parafulva* strain that degrades PFOA, but did not identify any of the intermediates. More recently, Huang and Jaffé (2019) demonstrated the defluorination of PFOA by *Acidimicrobium* sp. A6, which is an autotrophic bacterium that uses ammonium ion or hydrogen as an electron donor and Fe^3+^ as an electron acceptor, and perfluorohexanoic acid was detected as a metabolite in pure cultures. Regardless of the starting substrate, the individual enzyme catalysed steps involved in PFAS degradation in microorganisms are not understood.

A recent review of PFAS biodegradation acknowledged the limitations of microbial metabolism and proposed that a combination of biodegradation and physicochemical methods may enable complete destruction of PFAS (Zhang et al. 2022). Solar/visible-light photocatalysis is emerging as one of the promising sustainable advanced oxidation processes for the treatment of wastewaters containing various pollutants. In this process, the semiconductor catalyst materials are activated by the absorption of UV and/or visible light. It results in the excitation of an electron (${e}_{\mathrm{CB}}^{-}$) to the conduction band of the semiconductor, leaving behind a hole (${h}_{\mathrm{VB}}^{+}$) in the valence band, which initiates the catalytic degradation process. Apart from the direct reaction of ${e}_{\mathrm{CB}}^{-}$ and ${h}_{\mathrm{VB}}^{+}$ with the pollutants, a significant fraction of these charge carriers react with the chemisorbed H_2_O and O_2_ molecules present on the catalyst surface, generating powerful reactive oxygen species (ROS) such as hydroxyl radicals and superoxide radicals leading eventually to the decomposition of the pollutants (Berger et al. 2005; Qian et al. 2019).

In recent research, focused particularly on developing new visible-light active photocatalysts for the degradation of PFAS, bismuth oxyiodide (BiOI)–based semiconductor materials have received greater attention than more traditional titanium-, zinc-, and indium-based photocatalysts (Paul Guin et al. 2022). The ability to absorb light in the visible region of solar spectrum and an increased mobility of photogenerated charge carriers by the induced dipoles of [Bi_2_O_2_]^2+^ slabs in BiOI increase its photocatalytic performance (Lin et al. 2022). However, to compensate the limitations related to the recombination of the charge carriers in BiOI, a number of methods have been taken up. Among them, an iodine deficient BiOI material is a recent and promising track (Wu et al. 2018). Iodine deficient visible-light active BiOI photocatalysts, containing both Bi_4_O_5_I_2_ and Bi_5_O_7_I phases, have been synthesised, and the degradation efficiency of such catalysts to degrade PFOA in water was investigated under simulated sunlight irradiation (Paul Guin et al. 2023). This work has shown that the iodine-containing mixtures convert PFAO through a combination of several parallel redox and radical reaction routes. A series of potential oxidant quenching studies conducted within have suggested that photogenerated high-energy electrons in the conduction band, electrons located at surface iodine vacancies, and superoxide radicals formed in the aqueous phase all contribute to the PFOA degradation reactivity. Interestingly, these materials do not have the appropriate valence band energy to generate the ubiquitous photocatalyst generated oxidant (OH radicals) but it should be noted that these have in the recent past been shown not to promote the degradation of PFOA (Javed et al. 2020; Xu et al. 2020).

Recently, Ding et al. (2021) demonstrated that very low concentrations of PFOA (0.5 ppm) could be degraded synergistically by a combination of photocatalysis and microorganisms from activated sludge. Immobilisation of a Bi_12_O_17_Cl_2_ photocatalyst and the microbial community onto polyurethane sponge improved degradation compared with the reactivity observed with the photocatalytic treatment alone: PFOA removal increased from 49 to 80% and defluorination increased from 33 to 60%.

In this paper, we explore the possibility of using photocatalysis and biocatalysis in a sequential manner to degrade high (100 ppm) concentrations of PFOA, which has not so far been reported in the literature, to our knowledge. For the first time, a fungus, *C. elegans*, was shown to partially biodegrade PFOA, and the combination of photocatalytic treatment over an iodine-deficient bismuth oxyhalide catalyst under simulated solar light, followed by incubation with the fungus, substantially improved degradation and defluorination of PFOA. Through analysis of the fluorometabolites formed, we propose a rationale for the improvement and a possible pathway for PFOA biodegradation in the fungus.

## Materials and methods

### Synthesis of photocatalyst

Equimolar (2.0 mmol) solutions of bismuth nitrate and potassium iodide were prepared in ethylene glycol. The latter solution was gradually added to the former solution with stirring. The resultant yellow-coloured solution was then transferred into a Teflon-lined autoclave and subjected to continued heating in a muffle furnace at 160 °C with a heating rate of 10 °C/min for 12 h. The resultant product was washed thoroughly with water and ethanol and the cake was dried at 80 °C. An aliquot of the dried sample was calcined at 400 °C, with a temperature ramp of 10 °C/min, for 2 h yielding the final product.

### Photocatalytic degradation of PFOA

The photocatalyst (0.5 g/L) was dispersed well in 10 mL of 100 ppm PFOA solutions in 50-mL glass vials that were kept in the dark for 1 h to reach an adsorption–desorption equilibrium. The pH of 1 ppm PFOA solution increased from 6.3 to 7.3 ± 0.2 after addition and dispersion of the iodine-containing materials. The vials containing PFOA solutions were then irradiated using an Atlas Suntest™ CPS + solar simulator with a 700-W Xenon lamp for different time intervals. After irradiation, the photocatalysts were separated by centrifugation and the supernatants were stored for further studies. A control experiment was conducted without the addition of any catalyst. The degradation (%) of PFOA was monitored using GC–MS by measuring its concentrations before and after photocatalysis (Eq. 1).

1 $$\mathrm{Degradation\;}(\mathrm{\%})\mathrm{\;of\;PFOA\;}=\frac{{C}_{0}- \, {\text{C}}_{\text{t}}}{{\text{C}}_{0}}\times 100$$

${C}_{0}-{\text{C}}_{\text{t}}$ signifies effective degradation of PFOA where, *C*_0_ and *C*_*t*_ are the initial and final concentrations of PFOA before and after irradiation, respectively, and *t* is the time of photoirradiation.

Details of the GC–MS methods used to determine PFOA removal (and formation of the intermediates produced along the degradation pathways) as well as details of the measurement of F^−^ produced following photocatalysis (as a measure of complete degradation) are outlined below.

### Fungal cultivation and biological degradation of PFOA

*Cunninghamella elegans* DSM1908 (CEL) was cultured according to the procedure described in Khan and Murphy (2021b). The fungus was initially streaked on Sabouraud dextrose agar plates and incubated at 28 °C for 4–5 days to grow the mycelia. The inoculum was prepared by homogenising the entire contents of the agar plates in 100 mL sterile water using a hand blender. To grow suspended fungal cultures, 45 mL autoclaved Sabouraud dextrose broth was inoculated with 5 mL fungal inoculum in 250 mL Erlenmeyer flasks, which were incubated at 28 °C for 72 h with 150-rpm agitation. Culture aliquots (10 mL) were transferred to 50-mL Erlenmeyer flasks and 1 mg of PFOA (100 ppm) was added; the flasks were incubated at 28 °C with 150 rpm agitation for different time intervals (12–60 h). Control experiments were conducted in which either the fungus was incubated in the absence of PFOA or the substrate was incubated without fungus. For the combined photodegradation-biodegradation experiments, the reaction mixture following photocatalysis was lyophilized overnight, and the residue was added to fungus as described above and incubated for 48 h. The fungal biomass was harvested by centrifugation at 8000 rpm, and the culture supernatants were extracted with 50 mL ethyl acetate using a 250 mL separating funnel. The organic layers were collected, the solvent was removed under reduced pressure, and metabolites were analysed by GC–MS and LC–MS. Quantification of PFOA degradation was quantified as previously described (Eq. 1).

### GC–MS analysis

Dried residue remaining after solvent evaporation was collected in 2-mL glass vials and 30–50 µL MSTFA was added. The vials were heated at 50 °C for 2 h and 1 mL dichloromethane was added (Khan and Murphy 2022). Samples (1 μL) were introduced with a splitless injection into an Agilent 7890B/5977A Gas Chromatograph/Mass Selective Detector equipped with a HP-5MS capillary column (30 m × 0.25 mm × 0.25 μm). The initial GC oven temperature was set at 55 °C for 4 min then raised at a rate of 10 °C/min to 300 °C, and the MS was operated in the scan mode (*m/z* 50–650).

### LC–MS analysis

The samples for LC–MS analysis were prepared in 2-mL glass vials and 0.5-µL sample volume was injected into an Agilent 6546 QTOF Mass Spectrometry system, equipped with an Agilent 1260 Infinity Prime II LC installed with a Zorbax Eclipse Plus C18 RRHD column (2.1 × 50 mm × 1.8 µm). The mobile phase consisted of 65% acetonitrile and 35% water with 0.1% trifluoracetic acid pumped at a flow rate of 0.5 mL/min. MS acquisition was carried out with an AJS (Agilent Jet Stream) ESI source equipped in Agilent 6546 system. The MS analysis was carried out in the negative mode with the following source conditions: drying gas temp 150 °C at 8 L/min, sheath gas 150 °C at 11 L/min, capillary voltage 4000 V, nozzle voltage 2000 V, and fragmentor voltage 100 V. The data analysis of extracted ion chromatograms (EICs) and their corresponding MS spectra was carried out using Agilent MassHunter Qualitative Analysis 10.0 software. MS data were searched via compound matching using the Agilent FBF (find-by-formula) algorithm, matching for singly charged monomeric ion species for common ions such as [M-H]-.

### Fluoride ion estimation

The free fluoride ion concentrations produced in photocatalytic degradation and biodegradation experiments were determined by fluoride ion selective electrode (Thermo Orion model 290) as described previously (Khan and Murphy 2021a). Two NaF standards: 0.2 mM and 2 mM, were prepared for electrode calibrations. Fluoride ion was measured in culture supernatant and in cell lysate after sonication (Sonics 130 W ultrasonic processor with a CV-18 probe) at 50% amplitude, 1 s pulse, on and 1 s pulse off for a total of 15 min on ice; 1 mL was added to 1 mL H_2_SO_4_ (1 M) and 8 mL buffer (0.5 mM trisodium citrate and 0.5 mM KNO_3_) to estimate free F^−^ ion.

## Results and discussion

### Combined photocatalyst-fungus treatment effectively degrades high concentrations of PFOA

**Fig. 1:**
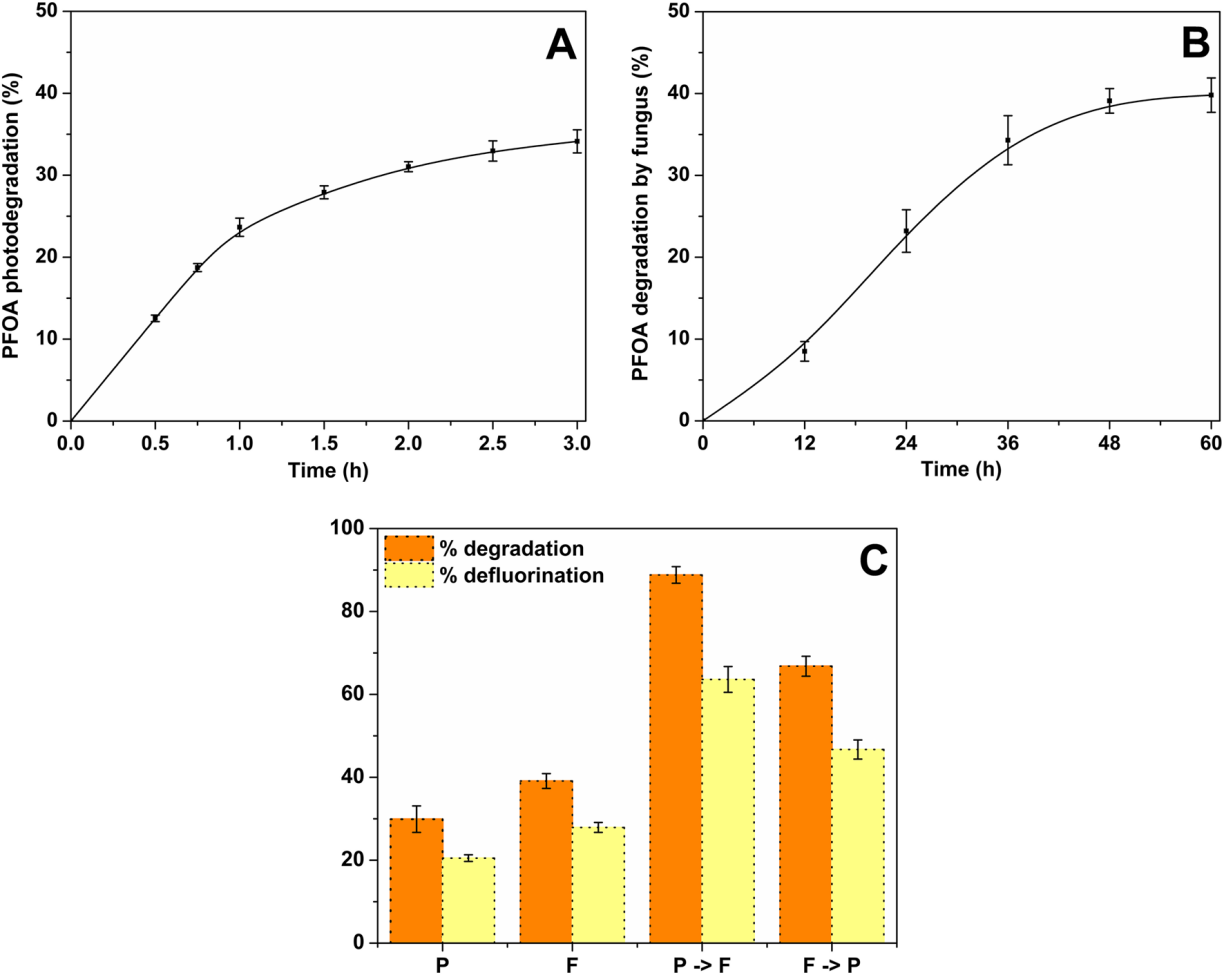
Degradation of PFOA by photocatalysis (**A**), fungal biodegradation (**B**), and combinations of both (**C**). P photocatalyst, F fungus, P → F photocatalyst (2 h) followed by fungus (48 h), F → P fungus followed by photocatalyst. Degradation was measured using the GC–MS peak areas of PFOA and metabolites, and defluorination was calculated from fluoride ion concentration measured by ion selective electrode

The photocatalyst and fungus were separately incubated with PFOA (100 ppm) to establish its degradation with each treatment. The concentration of the substrate was measured using GC–MS and fluoride ion was measured via an ion selective electrode. Treatment of 100 ppm PFOA with the photocatalyst for 3 h resulted in approximately 35% overall degradation, with 20% defluorination (Fig. 1). The degradation is less than that reported with a BiOI@Bi_5_O_7_I composite, which degraded approx. 80% of 15 mg/L PFOA albeit over a longer reaction time of 6 h (Wang et al. 2020). The profile of degradation shown in Fig. 1 suggests that a longer reaction time would not result in a very much improved outcome; thus, the lower reactivity is probably due to a catalyst poisoning effect rather than any kinetic effect. In any case, our previous work using this catalyst has suggested that a combination of electrons in the valence bands of the catalysts or those located at iodine vacancies (shown to be active through appropriate AgNO_3_ scavenging experiments) is an important reaction mediator and the holes remaining in the valence band following photoexcitation have only limited reactivity (Paul Guin et al. 2023). There is also a role for O_2_^●−^ which can be produced from conduction band electrons reacting with O_2_ (as shown by scavenging experiments using para benzoquinone). Interestingly, the photogenerated valence band holes are not sufficiently oxidising to generate OH radicals (the ubiquitous oxidant in aqueous phase photocatalysis).

Having generated a reactivity baseline for the photocatalyst alone the next step was to determine the reactivity of the fungus. The *C. elegans* performed similarly to the photocatalyst, with 40% degradation and 30% defluorination after 48 h (Fig. 1). Although this fungus was previously shown to degrade the related molecule 6:2 fluorotelomer alcohol (Khan and Murphy 2022), this was the first observation of degradation of PFOA in any fungus.

Sequential treatment of PFOA with the photocatalyst and fungus demonstrated a marked improvement in the degradation and defluorination of PFOA. The best outcome was observed with a photocatalytic treatment first (2 h) followed by incubation with the fungus (48 h), which resulted in an overall PFOA degradation of 90% with 60% defluorination (Fig. 1). In experiments where PFOA was treated with the fungus first, followed by the photocatalyst, 65% of the PFOA was degraded with 45% defluorination. This encouraging result points to a potential application to degrade high concentrations of PFOA in, for example, aqueous film-forming foam (AFFF). A combination of methods is likely necessary to completely degrade PFAS (Zhang et al. 2022; Banayan Esfahani et al. 2022), and here we have shown that combining two of the most environmentally sustainable methods currently being investigated, i.e. photodegradation and biodegradation, is much more effective that either method on its own.

### Identification of fluorinated products reveals the mechanism of improved degradation and a biodegradation pathway for PFOA

**Fig. 2:**
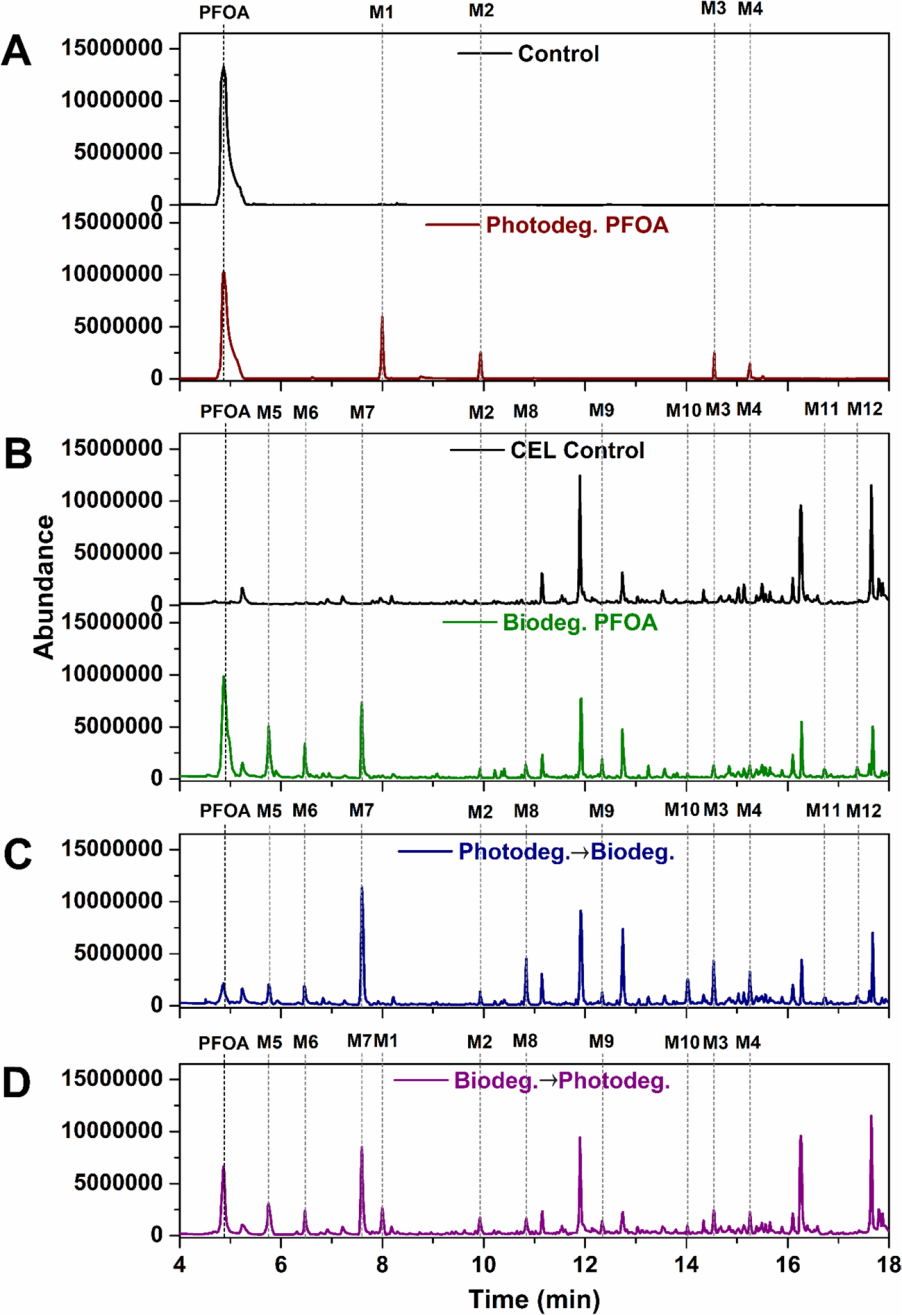
TIC from GC–MS analysis of extracts from treatment of PFOA with photocatalyst (**A**), fungus (CEL) (**B**), photocatalyst/fungus (**C**), and fungus/photocatalyst (**D**). The products and metabolites detected are indicated (M1–M10) and their mass spectra are given in the supplemental information

**Fig. 3:**
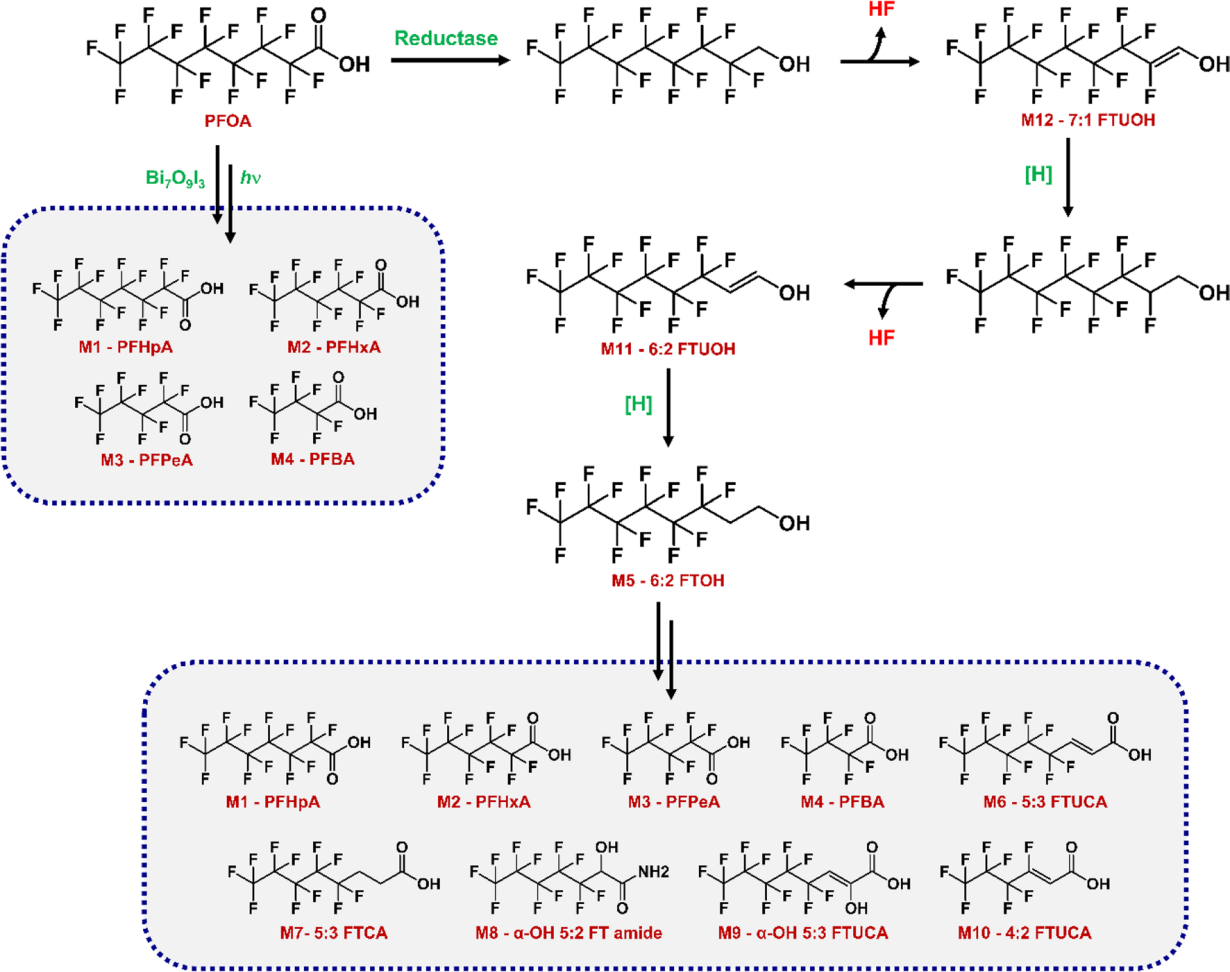
Summary of the proposed pathway of degradation following treatment of PFOA by photocatalysis and fungus

To explore the degradation mechanism of PFOA by the different treatments, the products from each experiment were analysed by GC–MS after derivatization by silylation (Fig. 2) and LC–MS (Table 1). Analysis of the products of the photocatalysis reaction showed that the silylated derivative of PFOA eluted from the GC after 4.8 min, and four photocatalytic degradation intermediates, labelled M1-M4, eluted after 8, 9.9, 14.5, and 15.3 min, respectively (Fig. 2A). Their mass spectra matched with those of silylated derivatives of perfluoroheptanoic acid (PFHpA, M1), perfluorohexanoic acid (PFHxA, M2), perfluoropentanoic acid (PFPeA, M3), and perfluorobutanoic acid (PFBA, M4), respectively (Fig. 3; S1–S5).

**Table 1:** Summary of LC–MS analysis of the products from PFOA photocatalytic and fungal degradation. The mass spectra are in the Supplemental Information

Metabolite	Empirical formula	CAS	Molecular weight	Exact mass	Prediction percentage
PFOA	C_8_HF_15_O_2_	335–67-1	414	413.9736	99.60
M1 (PFHpA)	C_7_HF_13_O_2_	375–85-9	364	363.9766	99.54
M2 (PFHxA)	C_6_HF_11_O_2_	307–24-4	314	313.9798	89.68
M3 (PFPeA)	C_5_HF_9_O_2_	2706–90-3	264	263.9831	90.88
M4 (PFBA)	C_4_HF_7_O_2_	375–22-4	214	213.9864	92.76
M5 (6:2 FTOH)	C_8_H_5_F_13_O	647–42-7	364	364.0115	96.05
M6 (5:3 FTUCA)	C_8_H_3_F_11_O_2_	-	340	339.9958	99.08
M7 (5:3 FTCA)	C_8_H_5_F_11_O_2_	914,637–49-3	342	342.0116	99.45
M8 (α-OH 5:3 FTUCA)	C_8_H_3_F_11_O_3_	-	356	355.9904	87.20
M9 (α-OH 5:2 FT amide)	C_7_H_4_F_11_NO_2_	-	343	343.0075	96.41
M10 (4:2 FTUCA)	C_6_H_2_F_8_O_2_	-	258	257.9927	89.42
M11 (6:2 FTUOH)	C_8_H_3_F_13_O	-	362	361.9779	85.71
M12 (7:1 FTUOH)	C_8_H_2_F_14_O	-	380	380.0154	87.86

The metabolites generated after 48-h fungal treatment of PFOA were also analysed by GC-and LC–MS (Fig. 2B; Table 1). Eleven compounds were detected (M2–M12), of which M2–M4 and M6–M10 are known metabolites of 6:2 FTOH (M5) biodegradation in the fungus (Khan and Murphy 2022). The detection of 6:2 FTOH as an intermediate provides some indication of the initial steps of PFOA biodegradation in *C. elegans*. The loss of fluorine without the loss of any carbon atoms strongly suggests that fluorine is lost as HF, and for this to happen, the PFOA must first be reduced to the alcohol. This is supported by the presence of two previously unobserved metabolites, M11 and M12, that can be tentatively identified as 6:2 fluorotelomer unsaturated alcohol (6:2 FTUOH, M11) and 7:1 FTUOH (M12) based on their mass spectra (Table 1; S1-S13).

A proposed pathway for the degradation of PFOA is shown in Fig. 3. Reduction of PFOA might be catalysed by a carboxylic acid reductase (CAR), which is known in fungi such as *Neurospora crassa* (Stolterfoht et al. 2018), although none has yet been detected in *C. elegans*. Alternatively, the substrate could be converted to the CoA ester and reduced to the alcohol by a fatty acyl CoA reductase. This would yield an aldehyde that could be reduced further to the alcohol, from which HF could be formally eliminated yielding M12. Reduction of this intermediate followed by loss of HF would yield M11, which in turn could be reduced to give 6:2 FTOH (M5). The specific enzymes involved in defluorination of fluorotelomer alcohols have only been speculated upon, for example, Wackett (2022) suggested that lyase might catalyse such a reaction, and Li et al. (2016) proposed that CYP-catalysed oxidation of 8:2 FTOH resulted in formation of an unstable aldehyde product that spontaneously eliminated HF. Our previous experiments indicated that CYP activity is crucial for the degradation of FTOH in *C. elegans* (Khan and Murphy 2022).

The most prominent metabolite observed from the fungal degradation of PFOA is 5:3 FTCA (M7), which was also the most prominent in previous studies with fungi incubated with 6:2 FTOH (Tseng et al. 2014; Khan and Murphy 2022). Furthermore, this compound was shown to be an inhibitor of fluorotelomer alcohol degradation in *C. elegans* (Khan and Murphy 2022).

Figure 2C shows the chromatogram measured from extracts after photocatalytic degradation followed by fungal treatment. As expected, the concentration of the substrate is substantially lower after either of the individual (photocatalysis or fungal) treatments and lower than the experiment in which the fungal treatment was applied prior to subsequent photocatalysis (Fig. 2D).

The peak of the 5:3 FTCA (M7) is still the most prominent after the photocatalysis followed by fungal treatment, but relative changes in the concentrations (as measured by peak heights) of other products are also apparent: those for M5 and M6 are lower compared to what was observed following fungal treatment only, whereas concentrations of M3, M4, M8, and M10 are all higher. M1 was not observed after the combined photocatalysis-fungal treatment. The likely explanation for the increased overall degradation with photocatalysis followed by fungal treatment of PFOA is that the main photocatalytic product (M1) is further metabolised by the fungus yielding a smaller chain carboxylic acid such as M3 and fluoride ion and avoiding the production of the inhibitory 5:3 FTCA. At the same time, the remaining PFOA is biodegraded to the other fungal metabolites, including 5:3 FTCA, through the routes described above. The accumulation of 5:3 FTCA eventually inhibits further catabolism, but because the concentration of PFOA and potential 5:3 FTCA precursors was initially lower (following the photocatalysis), biodegradation is prolonged compared with the fungal treatment only.

Conversely, when the PFOA is incubated with the fungus first and subsequently exposed to photocatalysis, 5:3 acid accumulates quicker and this, as before, inhibits biodegradation. That the subsequent photocatalytic treatment is relatively poor suggests either the 5:3 acid is also a poison for the photocatalytic reaction or more likely that the complex mixture of organic compounds present in the culture supernatant prevents effective photocatalysis, likely by scavenging the reactive species generated in this process, thus prohibiting these from reacting with the substrates of interest.

## Conclusions

In this paper, we demonstrated that sequential treatment of a relatively high concentration (100 ppm) of PFOA by a bismuth oxyiodide photocatalyst promoted by solar light, followed by the fungus *C. elegans*, resulted in 90% degradation with 60% defluorination. This was much better the reactivity observed in the individual treatments and a reverse fungal-photocatalytic treatment. Metabolite analysis indicated that initial photocatalytic degradation provides a decomposition route which avoids the accumulation of 5:3 FTCA, an inhibitor of fungal biodegradation, thereby prolonging the fungal catabolism of the photocatalytic products and the remaining PFOA. This method has potential for the sustainable remediation of environments contaminated with this, and other, PFAS. However, work is needed to optimise conditions to ensure complete degradation of PFOA and it might be anticipated that PFAS present in other matrices may not be as readily degraded as the pure compound used in this study owing to the presence of interfering or inhibitory substances.

## Supplementary Information

Below is the link to the electronic supplementary material.

Supplementary file1 (DOC 28442 kb)

## Data Availability

The datasets used/analysed in the study are available from the corresponding author on reasonable request.
