# Supplementary material for: Enhanced removal of perfluorooctanoic acid with sequential photocatalysis and fungal treatment
Source: Environ Sci Pollut Res Int. 2023 Jul 20;30(39):91478–86. doi: 10.1007/s11356-023-28588-5 (PMC10439853; doi:10.1007/s11356-023-28588-5)
Supplement: Supplementary file 1 — Supplementary file1 (DOC 28442 kb) [file 11356_2023_28588_MOESM1_ESM.doc]

**Supplementary Information**

**Enhanced removal of perfluorooctanoic acid with sequential photocatalysis and fungal treatment**

Mohd Faheem Khan,^a, 1^ Jhimli Paul Guin,^b,c, 1^ Ravindranathan Thampi,^b^ James A. Sullivan^c^ and Cormac D. Murphy^a^*

^a^ School of Biomolecular and Biomedical Science, University College Dublin, Belfield, Dublin 4, Ireland

^b^ School of Chemical and Bioprocess Engineering University, College Dublin, Belfield, Dublin 4, Ireland

^c^ School of Chemistry, University College Dublin, Belfield, Dublin 4, Ireland

^1^ Authors contributed equally to the manuscript

**Figure legends**

**Figure S1.** Mass spectra and structure of PFOA from GC-MS (silylated; M^+^ ­- 15) **(A)** and LC-MS **(B)**

**Figure S2.** Mass spectra and predicted structure of compound M1 from GC-MS (silylated) **(A)** and LC-MS **(B)**

**Figure S3.** Mass spectra and predicted structure of compound M2 from GC-MS (silylated) **(A)** and LC-MS **(B)**

**Figure S4.** Mass spectra and predicted structure of compound M3 from GC-MS (silylated) **(A)** and LC-MS **(B)**

**Figure S5.** Mass spectra and predicted structure of compound M4 from GC-MS (silylated) **(A)** and LC-MS **(B)**

**Figure S6.** Mass spectra and predicted structure of metabolite M5 from GC-MS (silylated) **(A)** and LC-MS **(B)**

**Figure S7.** Mass spectra and predicted structure of metabolite M6 from GC-MS (silylated) **(A)** and LC-MS **(B)**

**Figure S8.** Mass spectra and predicted structure of metabolite M7 from GC-MS (silylated) **(A)** and LC-MS **(B)**

**Figure S9.** Mass spectra and predicted structure of metabolite M8 from GC-MS (silylated) **(A)** and LC-MS **(B)**

**Figure S10.** Mass spectra and predicted structure of metabolite M9 from GC-MS (silylated) **(A)** and LC-MS **(B)**

**Figure S11.** Mass spectra and predicted structure of metabolite M10 from GC-MS (silylated) **(A)** and LC-MS **(B)**

**Figure S12.** Mass spectra and predicted structure of metabolite M11 from GC-MS (silylated) **(A)** and LC-MS **(B)**

**Figure S13.** Mass spectra and predicted structure of metabolite M12 from GC-MS (silylated) **(A)** and LC-MS **(B)**


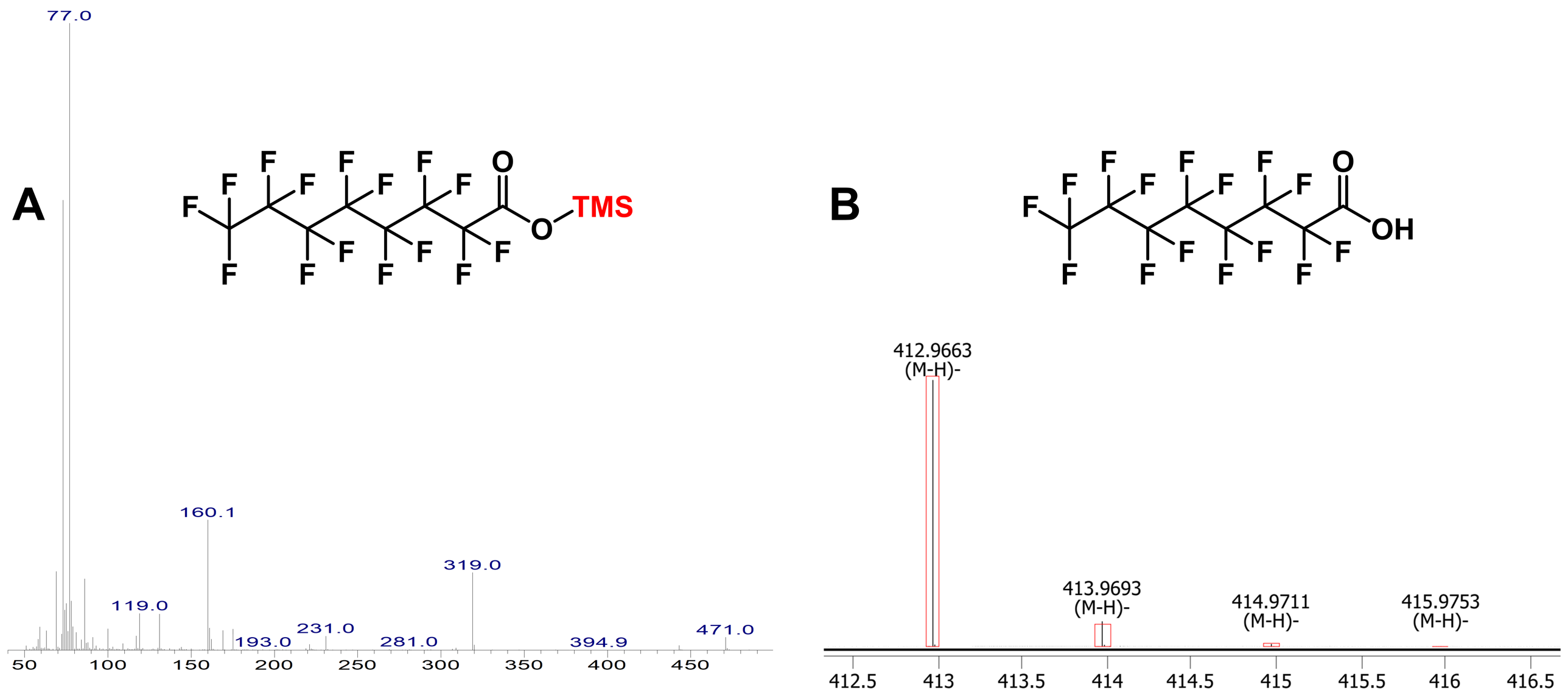


**Figure S1.** Mass spectra and structure of PFOA from GC-MS (silylated; M^+^ ­- 15) **(A)** and LC-MS **(B)**


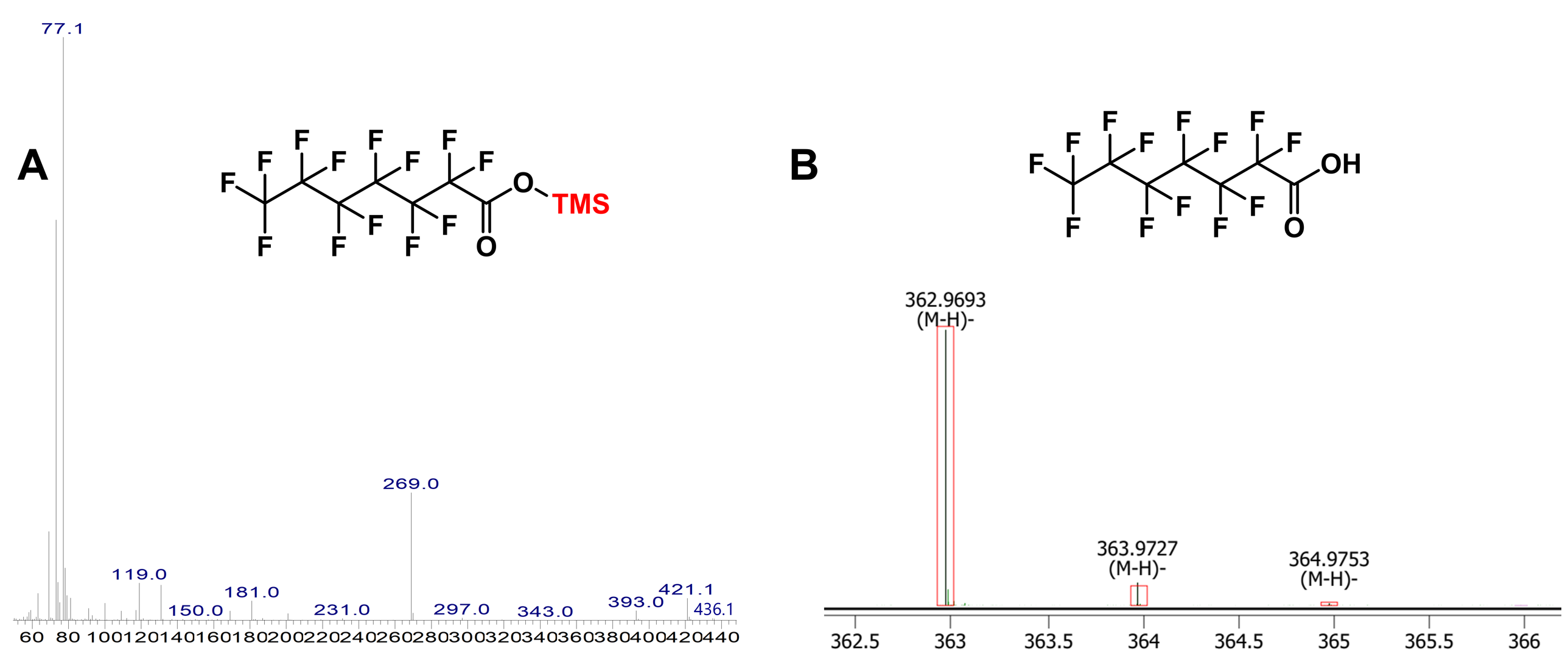


**Figure S2.** Mass spectra and predicted structure of metabolite M1 from GC-MS (silylated) **(A)** and LC-MS **(B)**


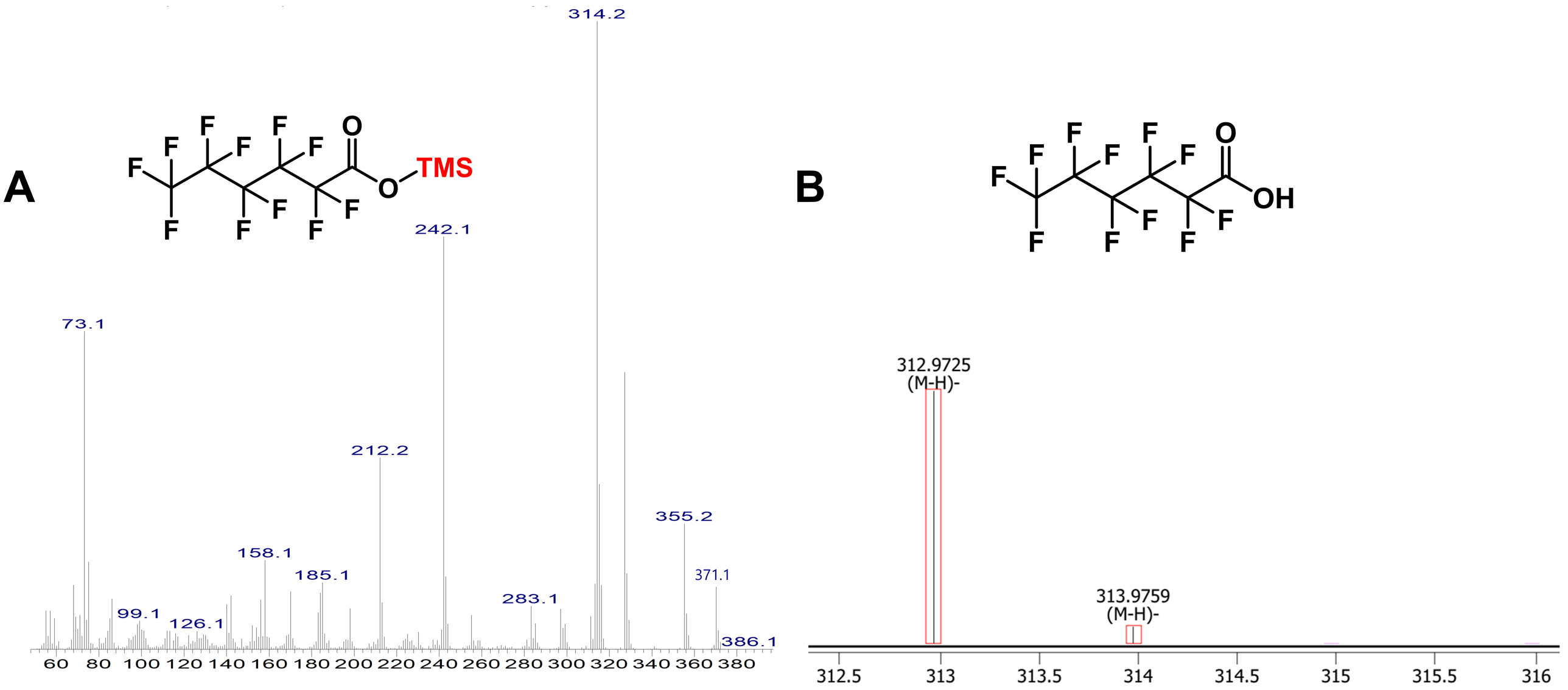


**Figure S3.** Mass spectra and predicted structure of metabolite M2 from GC-MS (silylated) **(A)** and LC-MS **(B)**


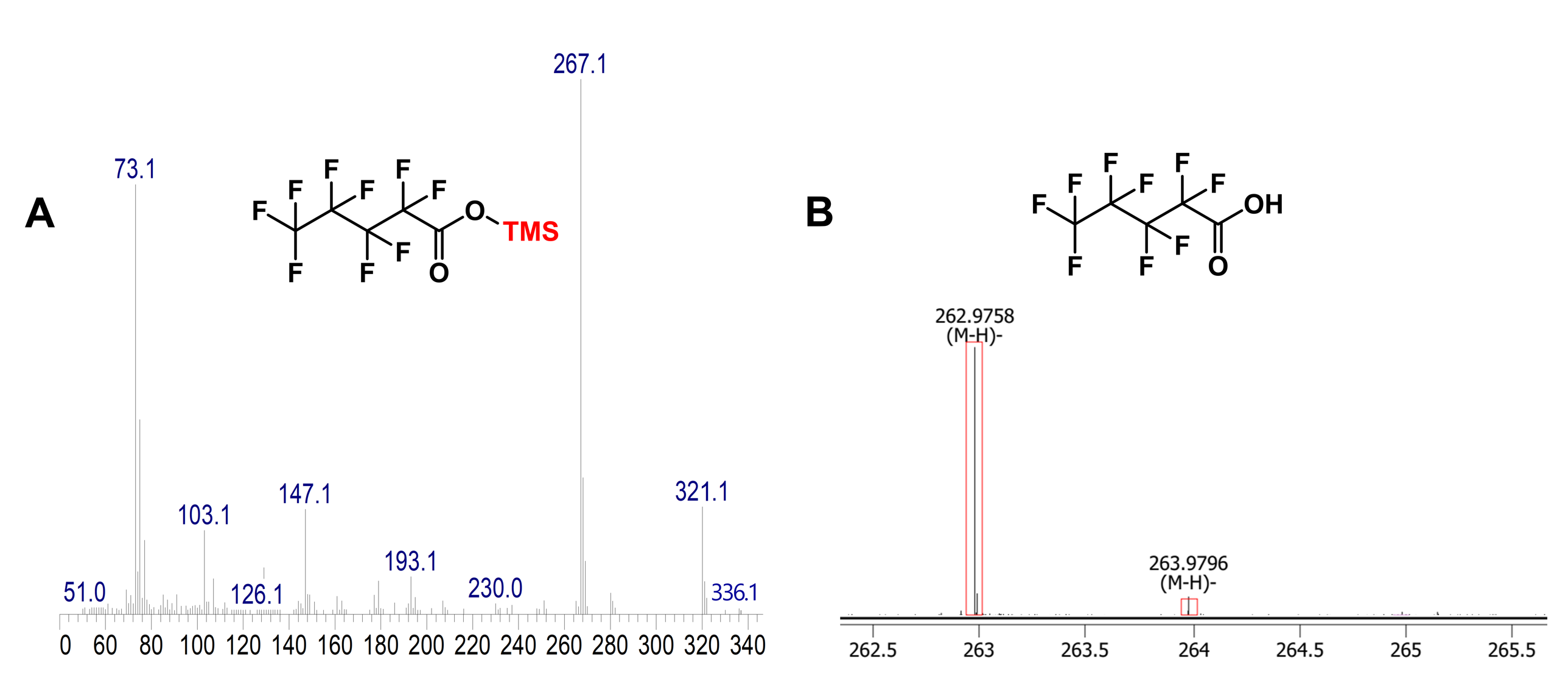


**Figure S4.** Mass spectra and predicted structure of metabolite M3 from GC-MS (silylated) **(A)** and LC-MS **(B)**


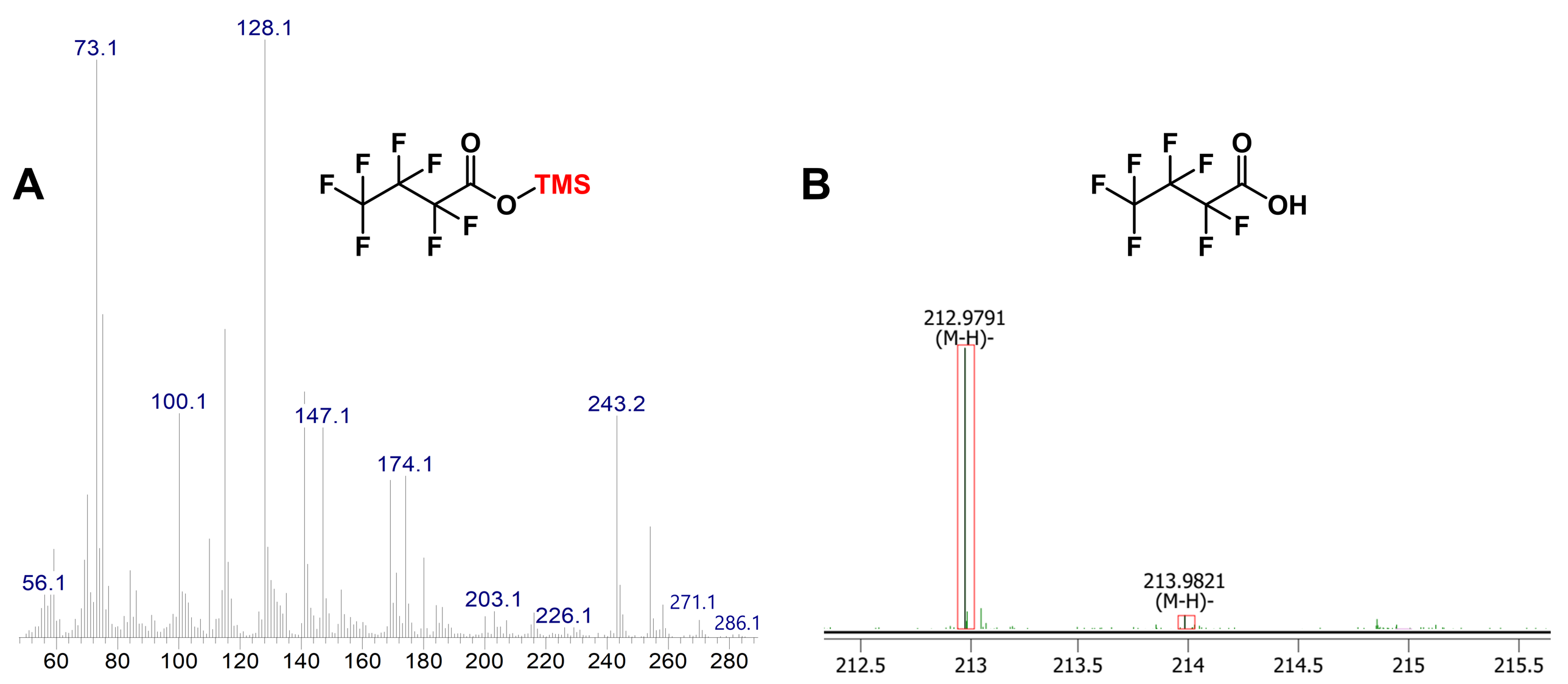


**Figure S5.** Mass spectra and predicted structure of metabolite M4 from GC-MS (silylated) **(A)** and LC-MS **(B)**


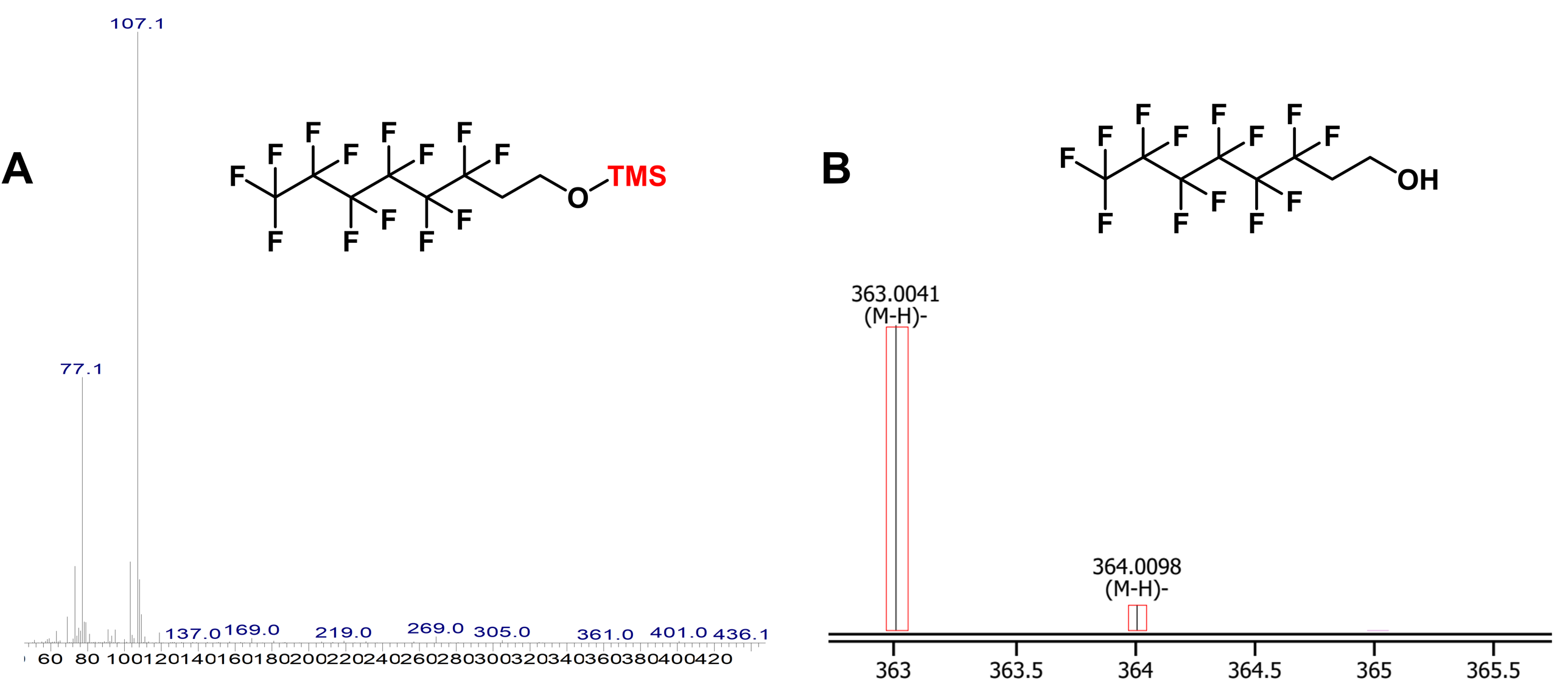


**Figure S6.** Mass spectra and predicted structure of metabolite M5 from GC-MS (silylated) **(A)** and LC-MS **(B)**


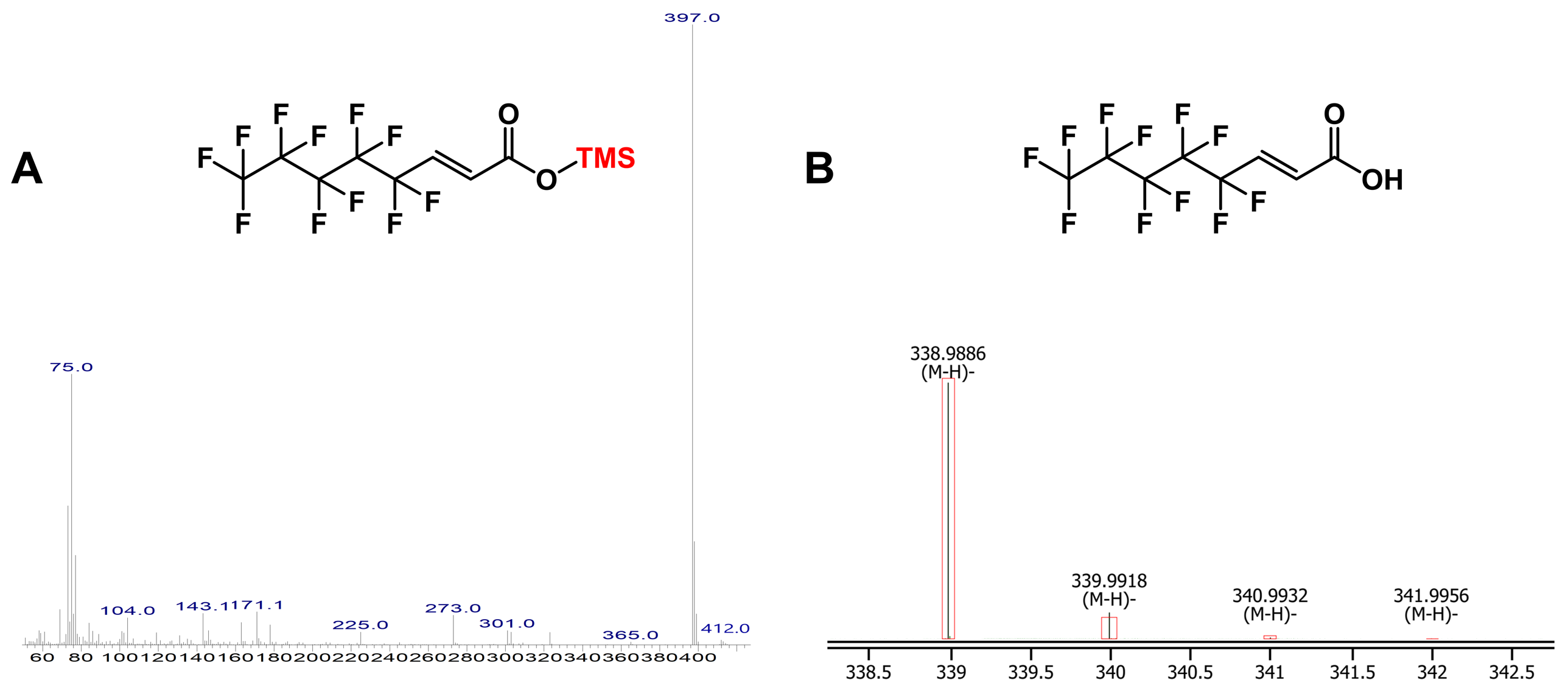


**Figure S7.** Mass spectra and predicted structure of metabolite M6 from GC-MS (silylated) **(A)** and LC-MS **(B)**


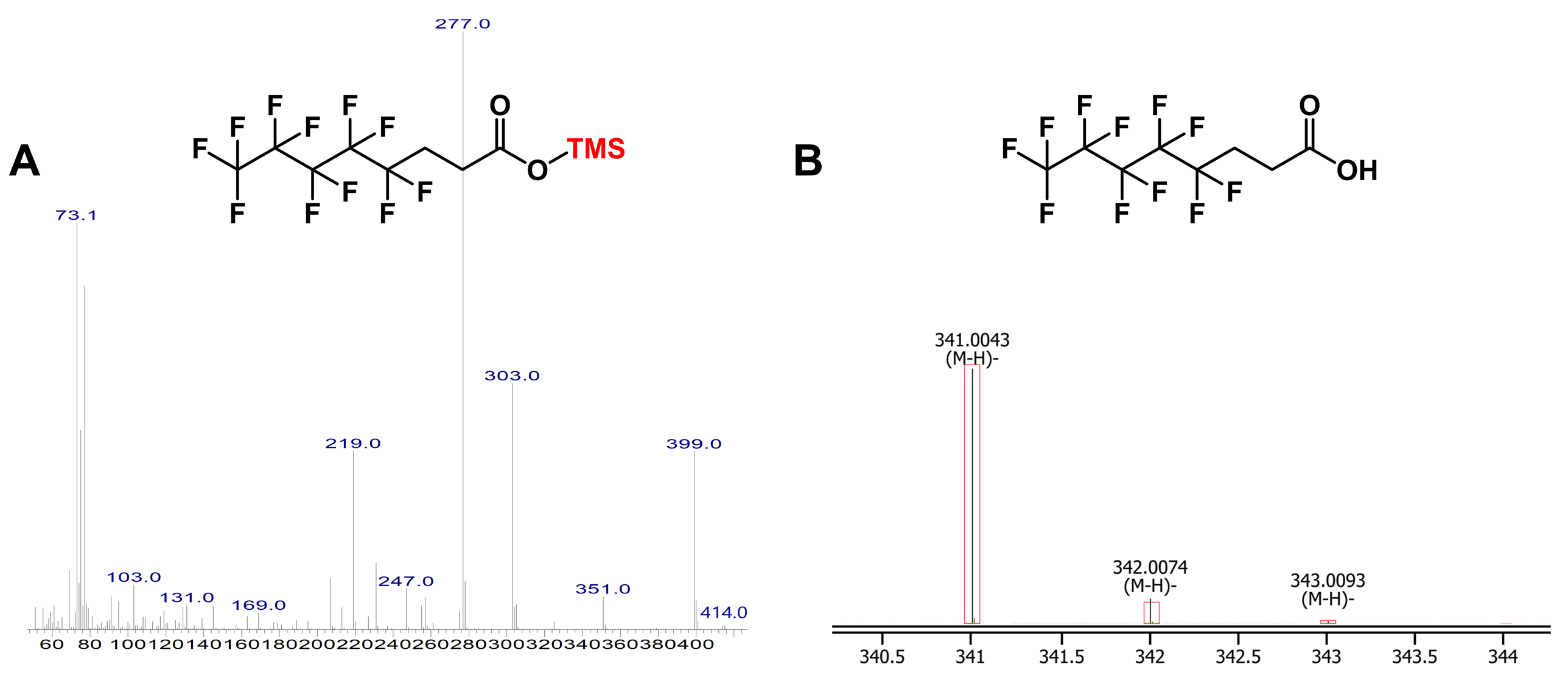


**Figure S8.** Mass spectra and predicted structure of metabolite M7 from GC-MS (silylated) **(A)** and LC-MS **(B)**


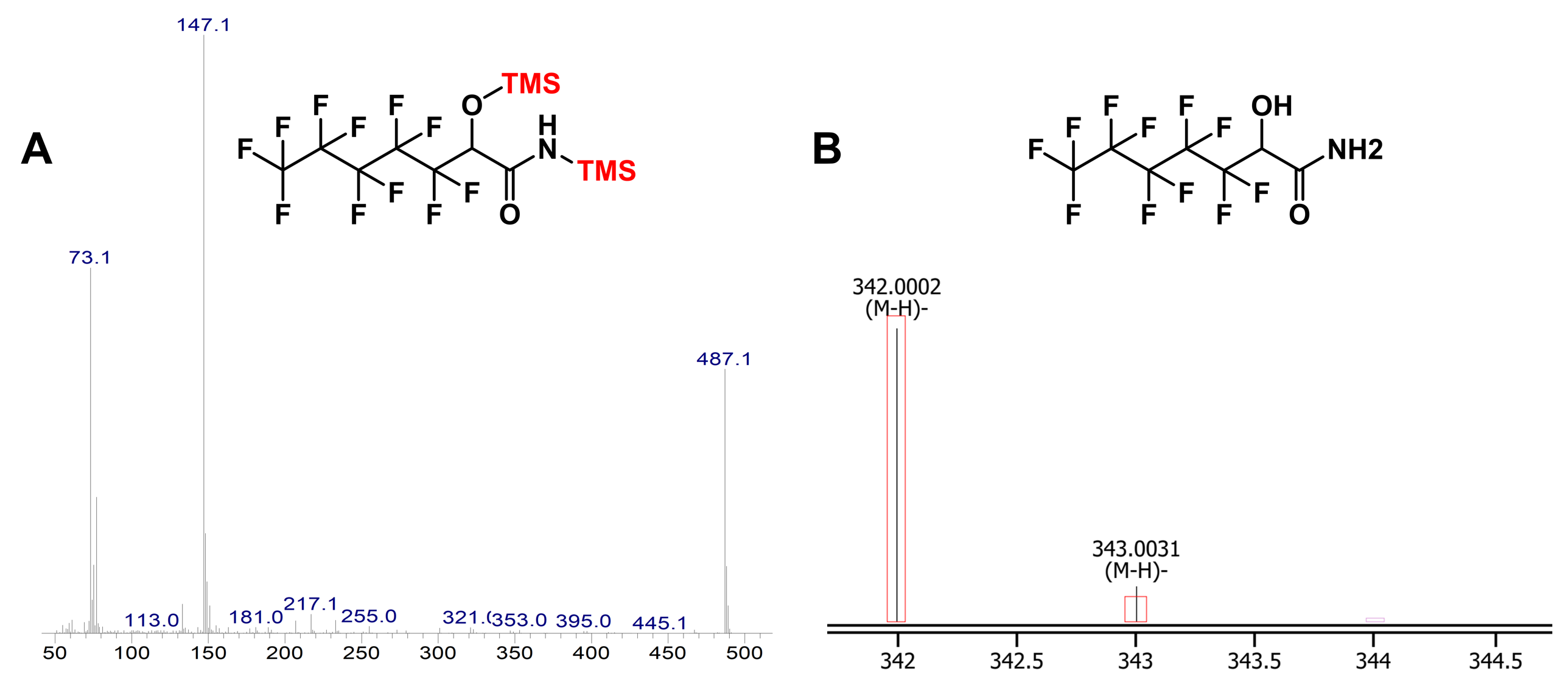


**Figure S9.** Mass spectra and predicted structure of metabolite M8 from GC-MS (silylated) **(A)** and LC-MS **(B)**


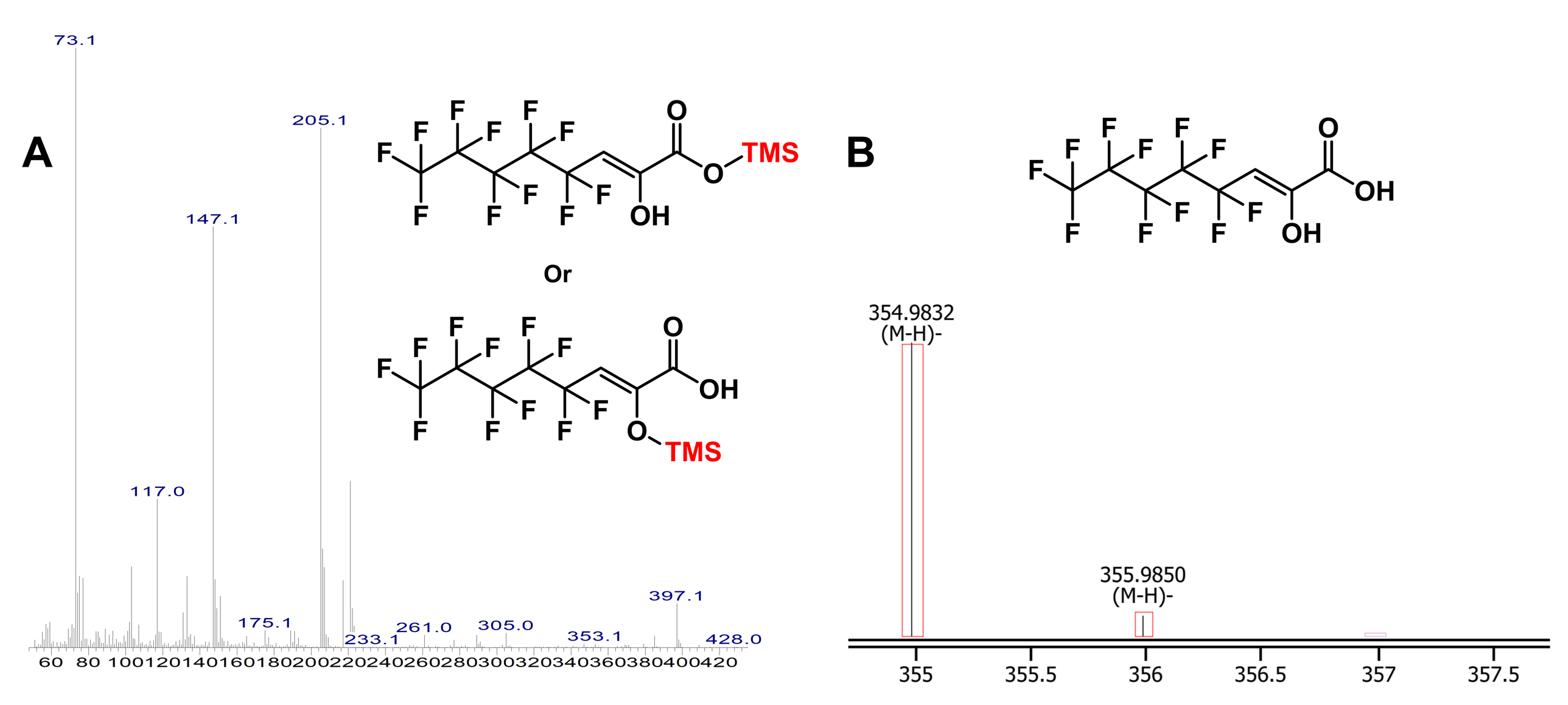


**Figure S10.** Mass spectra and predicted structure of metabolite M9 from GC-MS (silylated) **(A)** and LC-MS **(B)**


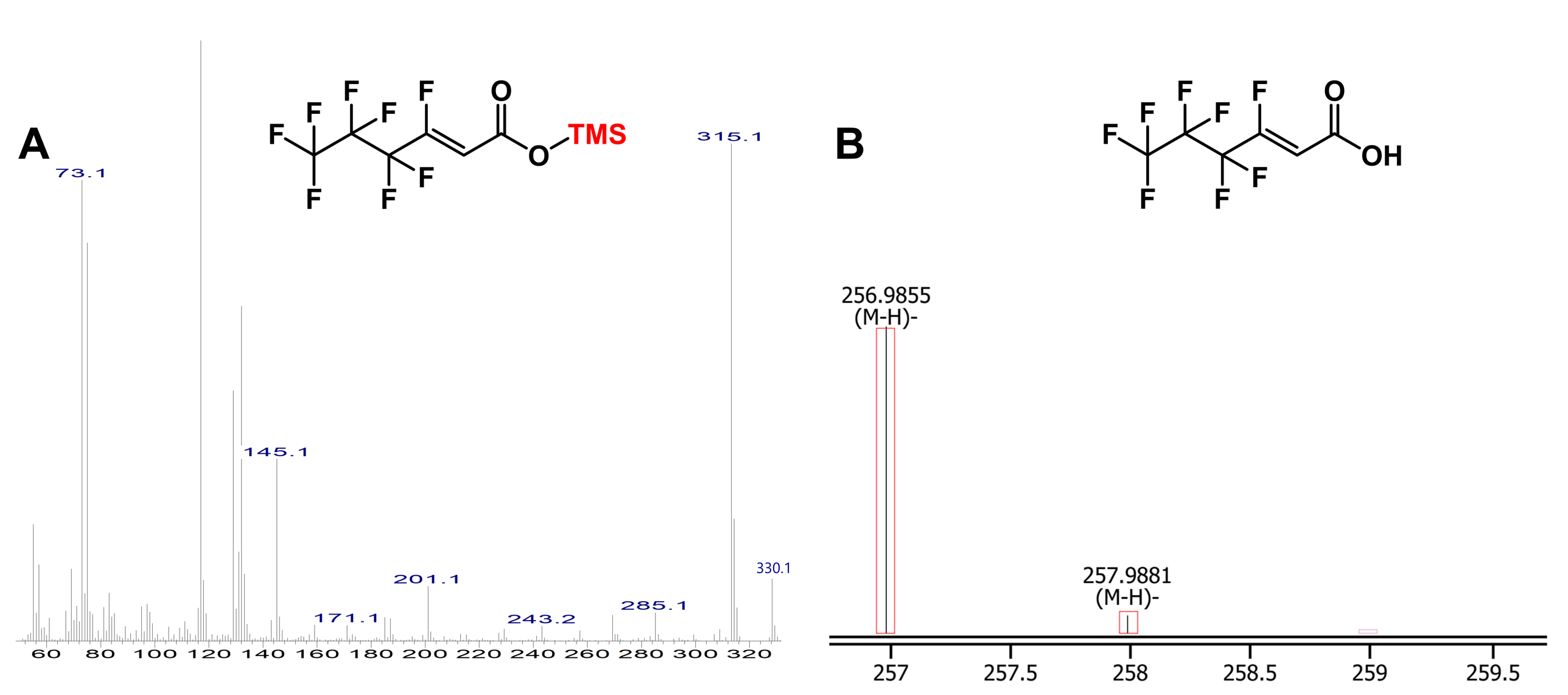


**Figure S11.** Mass spectra and predicted structure of metabolite M10 from GC-MS (silylated) **(A)** and LC-MS **(B)**





**Figure S12.** Mass spectra and predicted structure of metabolite M11 from GC-MS (silylated) **(A)** and LC-MS **(B)**


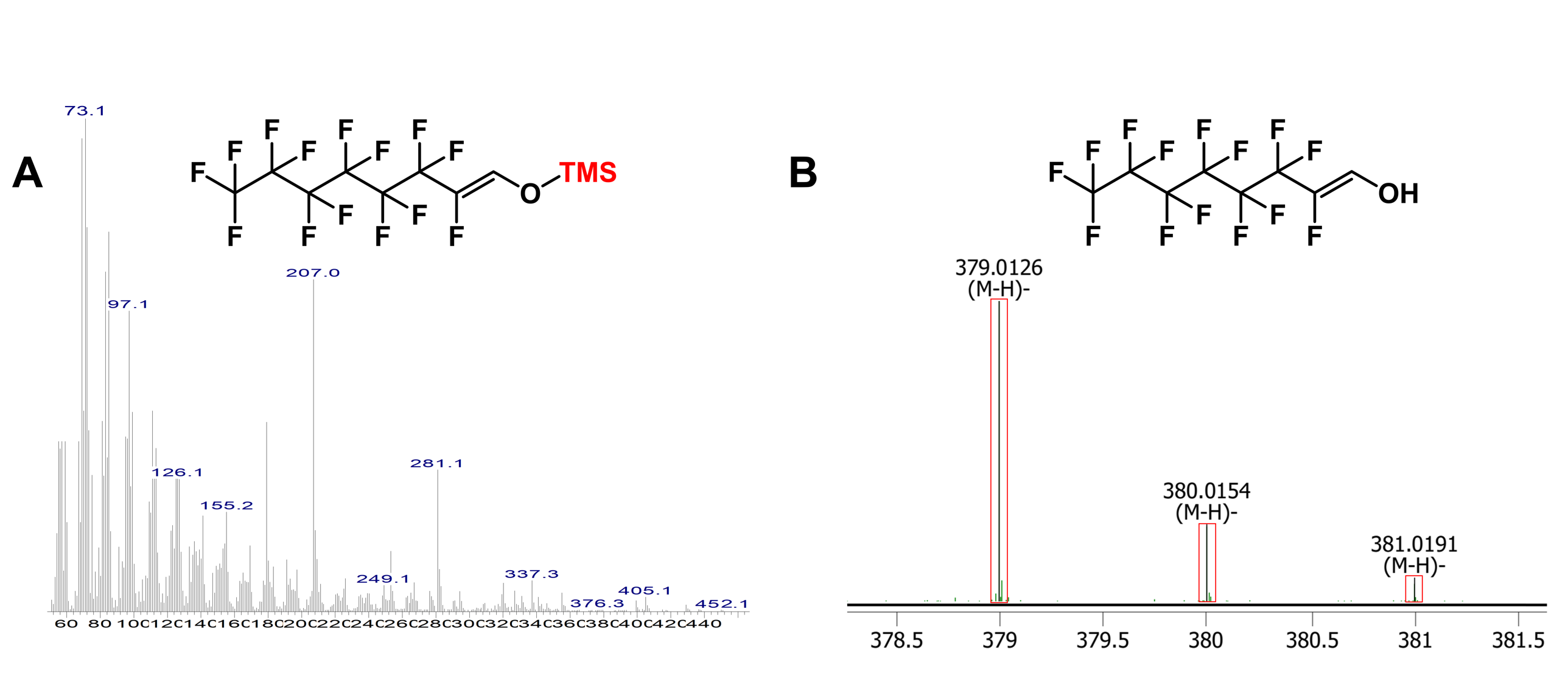


**Figure S13.** Mass spectra and predicted structure of metabolite M12 from GC-MS (silylated) **(A)** and LC-MS **(B)**
